# Supplementary material for: Knowledge, attitudes and practices towards emergency contraceptive pill use among women of reproductive age in Lira District, Northern Uganda: A cross-sectional study
Source: PLoS One. 2026 Mar 26;21(3):e0346151. doi: 10.1371/journal.pone.0346151 (PMC13020816; doi:10.1371/journal.pone.0346151)
Supplement: Attachment — Submitted filename: Reviewer cooments (AutoRecovered).docx [file pone.0346151.s002.docx]

| Reviewer #1: |  |
| --- | --- |
| Line 37 and 106 are contradictory. Authors need to clarify and state which of the dates is correct | Thank you for this observation the authors have corrected this contradicting statement |
| Line 38, 130,133,142,197 and 198 should be reviewed by the authors. Authors indicated that they we they going to recruit 600(indeed line 38 says that 600 were recruited), yet using the multi stage sampling to select the participants, 585 were selected and interviewed yet the authors still mentions a response rate of 97.5% and in another breath indicates that all the participants contacted agreed to the interviewed. Authors need to strongly look at this again. | We apologise for confusion the 600 was the estimated sample size but 585 were recruited for the study |
| Line 52: Authors should be specific and indicate what component of the marital status, attitude or the knowledge that influences the use of the EC. eg is it the unmarried or the married??? | This has been specified to the un married |
| Line 91-92,111:Can the authors report/describe contraceptive use (prevalence) instead of contraceptive non-use as stated in this paper | That you for this observation, this has been corrected as advised |
| Line 94-95: why do you select HIV population? Better to give the prevalence in the general population first. If that is not known, then the authors should say so. | This was the only study we came across in lira district that has information about unwanted pregnancy. Never the less we decided to remove this and noted it is unknown |
| Line 114- Much useful information with respect to this study should rather be women in reproductive age and not all women as stated by the authors. They should have a look at it again if the information exist. | This has been adjusted as advised. Thank You |
| Line 122: Did the authors include women who were already on regular effective contraception? that will obviously give different results which should be interpreted differently. Did we include those who were on regular contraception but had missed/delayed the next dose for which they could be at risk of unwanted pregnancy and hence would be candidate for EC? Authors should clarify these because they have implications on the findings. They can not be lumped together. Again, if a person is looking for pregnancy, then obvious EC does not come in all. The authors should be much clearer on the eligibility criteria. | Thank you for this insightful observation. We agree that distinguishing between women on regular contraception and those not using it—or using it irregularly is crucial in interpreting EC use data accurately. We have now clarified our eligibility criteria and explained how we addressed this issue in our study design and analysis. |
| Line 146: Where did you pre-test the tool?? | The tool was pretested in Kole district and this information has been added to the data collection tools |
| Line 155- suggested to authors that it should be 'after unprotected sexual intercourse' | Thank you for this suggestion this had been included in the write up |
| Line 167-167: frame this statement properly..... it looks like an important conjunction 'and' is missing somewhere and makes the it hanging | This has been addressed thank you |
| Line 200: kindly refer to the study participants as women and not girls | Thank you for this observation this has been addressed |
| Line 253: what was the duration for which the use of EC was studied (was it the previous one month as the present study or different?) | Thank you for this clarification EC use was assed for the previous month |
| Line 255: what was the setting here, these should come out......was this also in Uganda or the northern part of Uganda as well?? | Thank you for this observation this has been addressed |
| Line 259: was this also assessed over the previous month? what was the inclusion and exclusion criteria; it is likely to affect this in addition that this was not general community-based population | This was not assed over the period of one month |
| Line 273: was this study also in Uganda? and could the study population and educational levels accounted for it??? | Thank you for the observation. This has been clarified in the write up |
| Line 274: your discussion around this point is very unclear....what exactly do authors want to convey?? | Thank you for this observation the paragraph has been re written to clarify the issue |
| Line 280:to what information, what platform exist in Uganda that promote accessibility to this information, authors need to bring this out | Thank you for this concerned this has been addressed |
| Line 282-288:repeation of paragraph 270-276 to this section ..........it is repetition of the same information | Thank you for this observation this has been removed from the write up |
| Line 320: which study was authors referring to? Recommending that the authors report on the figures they are referring to. | Thank you for this observation. this has been corrected |
| Line 325: causality cannot be determined in cross sectional study....it may be associated but not causality as the authors put it. They should revise it. | This has been revised as advised thank you. |
| Line 332: Incomplete sentence. Authors should address it. | Thank you for this |
| Line 382-385: The reference is captured with block letters and it is not consistent with what the other references have been captured. | Tis has been corrected thank you |
| Table 2: With respect to age, which category did the authors put the 18-year-olds? and those who were 24 and 35 years.... need to re categorize properly. what informs this sub groupings, there appears to be no science behind it......could refer to the groupings used by your DHS or some better sub categorization. | We thank the reviewer for this important observation. We acknowledge that the initial age groupings lacked explicit justification and may have created ambiguity around boundary ages. We have now revised the age categories in accordance with the Uganda Demographic and Health Survey (UDHS) 2016 classification, which is widely recognized in reproductive health research. Specifically, we reclassified age into the following categories: 15–19, 20–24, 25–29, 30–34, 35–39, and 40–49 years. Each participant was assigned to a group based on completed age. This revision ensures comparability with national data and enhances the scientific rigor of our analysis. Table 2 and related text have been updated accordingly. |
| Authors should have a second look at the following listed paragraphs and address the applicable grammatical errors, typos, concordance of sentences, repetition of words, appropriate use of capital and small letters and fragmentation of words among others...55, 62, 107, 117,118,122,128,129,140,141,158,170,171,184,202,205,232,233,242,244,295,299,301 and 321. | Thank you for this observation the manuscript has been revised for all typos and errors |
| Reviewer 2 |  |
| Background: 1. Clarity and Grammar: |  |
| o Line 55: The sentence "Contraceptive use is a challenge globally are a public health challenge" is grammatically incorrect and redundant. Please revise for clarity. | Thank you for this observation this has been addressed |
| o There is inconsistency in quantitative data related to the number of women of reproductive age and the need for family planning. For example, figures on contraceptive use in Uganda are unclear (350 vs. 40). These data points need to be clarified or synthesized for coherence. |  |
| 2. Redundancy in References: o Reference [2] is repeated unnecessarily. It should be cited only once, ideally at the end of the relevant statement. | This has been corrected |
| 3. Transitions and Word Choice: o Line 60: The use of “However” is not contextually appropriate. Please revise the sentence to better reflect the logical flow. | Thank you for this observation the word However has been removed from the sentence |
| 4. Structure and Writing Style: o The background section would benefit from a thorough review by a native English speaker to improve clarity, formal tone, and scientific rigor. | This has been reviewed by the authors. Thank you |
| o Some paragraphs are verbose and could be synthesized into concise, scientifically written statements. This comment can be directed to the whole paper. | The manuscript was rewritten to address this comment |
| Methods: |  |
| 1. Variable Selection for Multivariate Analysis: o Authors need to describe the process used for selecting variables included in the multivariate model. It is unclear if the variables shown in the tables are the only ones included or if others were considered but excluded. Authors need to specify the process by which they selected the variables for the multivariable analysis. | The process by which variables were selected for the multivariate analysis has been added to the manuscript in the data analysis section |
| 2. Specific Variables: o The variable religion is mentioned in the methods but not presented in the results. Please include or justify its omission. | Although religion was initially considered during the development of the questionnaire, it was excluded from the final tool to maintain brevity and focus on variables with stronger theoretical relevance to emergency contraceptive use |
| o Consider discussing other relevant variables, such as maternal education and sexual behaviors, which could add depth to the analysis. | With respect to *maternal education*, our study captured the participants' level of education, which is already presented in the results (Table 2) and discussed as a factor associated with emergency contraceptive use.  We acknowledge that *sexual behavior variables*, such as the number of recent sexual partners or condom use, were not collected in this study. We recognize this as a limitation and have included a statement in the discussion to reflect this. We agree that incorporating such variables in future research would provide a more comprehensive understanding of emergency contraceptive use. |
| 3. Time Frame Definition: o The study focuses on contraceptive use in the last month. If women who used emergency contraception more than once but not in the past month were categorized as non-users, this needs to be clarified in the objectives. Distinguish between factors associated with general use versus use in the previous month. | Thank you for this observation |
| Results: 1. Table Presentation: |  |
| o The title of Table 5 appears misaligned or misplaced. Please correct formatting. | Thank you for this observation the title has been corrected |
| o In Table 2, the section on contraceptive pill use is unclear. Specify whether this refers to contraceptive pills in general or emergency contraceptive pills. If it is emergency contraception, ensure consistency in column headings. | This has been addressed in table 2 thank you |
| o I recommend presenting the results related to knowledge and attitudes before discussing the factors associated with the outcome. Specifically, the univariate and multivariate analyses should follow this order for better flow and clarity | The order of presenting the results is as follows:   1. Sociodemographic variables 2. Knowledge towards use of emergency contraceptive pill 3. attitude towards use of emergency contraceptive pill 4. factors associated with emergency contraceptive use |
| Discussion: 1. Relevance and Precision: o The discussion claims that individual factors, knowledge, and attitudes are associated with the outcome, but only marital status is included in the multivariate analysis. This discrepancy needs to be addressed. | Overall knowledge and overall attitudes are the ones associated with emergency contraceptive use.  These are composite variables |
| 2. Comparative Analysis: o The discussion compares prevalence rates with other studies but does not address whether the definition of emergency contraceptive use (e.g., limited to the last month) aligns across studies. This difference could significantly impact comparisons and must be discussed. | Thank you for this observation |
| 3. Causal Inferences: o The authors mention the importance of access to information but do not provide data supporting this claim. Conclusions should be based only on findings from the study and not unsupported assumptions. | We acknowledge that our study defined emergency contraceptive pill use as use within the *previous one month*, whereas some of the studies we compared our findings with assessed *lifetime* or *ever use*. This difference in definitions could contribute to discrepancies in reported prevalence rates. We have now revised the discussion section to clearly highlight this distinction and caution against direct comparisons where timeframes differ. |
| 4. General Writing: o The discussion requires polishing for better flow and coherence. Revise to align statements with the presented results and ensure logical arguments. | The discussion sections have been revised ad advised thank you |
| Final Notes: 1. Consistency in Tables: o Ensure all tables are correctly labeled and formatted. Double-check the alignment of titles and contents. | This has been addressed as advised thank you |
| 2. Language and Formatting: o The manuscript needs a detailed language review to improve grammar, syntax, and adherence to scientific standards | We reviewed the language in the manuscript as advised. Thank you |
