# Supplementary material for: Knowledge, attitudes and practices towards emergency contraceptive pill use among women of reproductive age in Lira District, Northern Uganda: A cross-sectional study
Source: PLoS One. 2026 Mar 26;21(3):e0346151. doi: 10.1371/journal.pone.0346151 (PMC13020816; doi:10.1371/journal.pone.0346151)
Supplement: Attachment — Submitted filename: Peer Review Comments.docx [file pone.0346151.s003.docx]

**Peer Review Comments – PLOS ONE Manuscript Review**

Dear Editors,

Thank you for the opportunity to review this revised version of the manuscript. While I recognize the importance of the topic and the efforts made by the authors to address previous feedback, I believe the manuscript still requires substantial revision before it can be considered for publication. Several methodological and reporting issues persist, and new concerns have emerged upon closer review. I outline these below in the spirit of constructive feedback, in line with PLOS ONE’s standards of transparency, scientific rigor, and reproducibility.

**Major Comments:**

1. **Clarity and Logic of Study Design**
   - The inclusion criteria should be described before the exclusion criteria for clarity and proper flow of the methodology section.
   - The secondary objective—identifying factors associated with emergency contraceptive use—is not clearly stated in the objectives. This should be made explicit in the introduction and reflected throughout the manuscript.
2. **Measurement and Cut-Off Clarity**
   - In line 167, the manuscript mentions that scores above 13 represent "good knowledge." However, this is confusing, as the phrasing implies that a score of 13 is neither good nor poor. Please clarify the cutoff and specify the direction of the scale (i.e., does a higher score indicate better knowledge or attitude?).
   - Additionally, it is not clear whether this cutoff was validated or arbitrarily chosen by the authors. If it is author-defined, this should be clearly acknowledged with justification.
3. **Sample Size and Response Rate**
   - Lines 199–200 state that the calculated sample size was 600, but only 585 participants were recruited. However, this does not constitute a response rate. A response rate refers to the number of respondents relative to the number invited or contacted. If only 585 were contacted, the study did not reach its intended sample size, and this should be transparently described.
4. **Results Structure and Flow**
   - The structure of the results section should better align with the stated objectives. First, describe the characteristics of the sample, followed by knowledge, attitudes, and emergency contraceptive use. Only then should results pertaining to associated factors (i.e., the secondary objective) be presented.
5. **Variables and Analyses**
   - In the "Study Variables" section (line 163), please explicitly list the variables considered for the multivariate analysis.
   - It is unclear which covariates were included in the final model. Please specify these in the results section when presenting the multivariable analysis.
6. **Terminology and Consistency**
   - Ensure consistent use of terms throughout the manuscript. For instance, the methods section uses “good knowledge vs poor knowledge,” but the results refer to “knowledgeable vs not knowledgeable.” Consistency is essential, particularly if the categories are author-defined.
   - Spelling and typographical errors should be corrected, such as "un married" (line 241) which should be "unmarried," and the double period on line 233.
7. **Table and Figure Presentation**
   - Table titles should appear directly above the corresponding tables. For example, Table 5 is mentioned in the text before it appears, creating confusion.
   - Table 4 is labeled as referring to knowledge, but the accompanying text addresses attitudes. This inconsistency should be corrected.
8. **Interpretation of Attitudinal Data**
   - Line 230 refers to neutrality regarding the effectiveness of emergency contraceptives. It is unclear whether this is measuring belief in efficacy or safety. The phrasing should be revised to clarify the item participants responded to (e.g., “The majority of respondents were neutral in response to the statement: ‘Emergency contraceptives are safe and effective.’”).
9. **Discussion and Framing**
   - The discussion begins by emphasizing usage and associated factors, but the primary objective is to assess knowledge, attitudes, and use. Please realign the discussion to reflect the stated goals.
   - The phrase “individual factors” (line 254) is used in the plural, yet only one factor (marital status) appears significant. This should be corrected.
   - Lines 271–286 repeat content from lines 251–270. Please revise to avoid redundancy.
10. **Conclusion and Bias Concerns**
    - The conclusion (line 297) suggests that non-use of emergency contraception is due to access limitations. This is an overgeneralization and could reflect bias. Alternative explanations—such as lack of perceived need, sexual inactivity, or personal preference—should be acknowledged.
    - Similarly, line 352 suggests that married women use regular contraception due to planned sexual activity. Yet, if your inclusion criteria excluded those using regular contraceptives, this reasoning may not apply.
11. **Ethical Reporting and Safety Discussion**
    - The manuscript seems to advocate for proactive and regular use of emergency contraception. If this recommendation is made, information about the safety of frequent use must be included, ideally in the introduction and discussion sections.
12. **Formatting and Language**
    - The acknowledgements section (lines 364–365) is incomplete.
    - Careful proofreading is required to correct grammatical errors and awkward phrasing throughout.

**Summary Recommendation:**

In its current form, the manuscript does not meet the methodological and reporting standards expected for publication in PLOS ONE. Significant revisions are necessary to enhance clarity, consistency, and rigor. I encourage the authors to address the issues outlined above in a thorough and transparent manner.

Sincerely,
