# Supplementary material for: Knowledge, attitudes and practices towards emergency contraceptive pill use among women of reproductive age in Lira District, Northern Uganda: A cross-sectional study
Source: PLoS One. 2026 Mar 26;21(3):e0346151. doi: 10.1371/journal.pone.0346151 (PMC13020816; doi:10.1371/journal.pone.0346151)
Supplement: Attachment — Submitted filename: Major Comments 2.docx [file pone.0346151.s004.docx]

Dear reviewers

We sincerely appreciate your time and effort in reviewing our paper. Your comments have been invaluable in improving the quality of our article. Thank you

| **Comments** | **Responses** |
| --- | --- |
| 1. Clarity and Logic of Study Design  The inclusion criteria should be described before the exclusion criteria for clarity and  proper flow of the methodology section | Thank you for reviewing our paper. This has been rewritten as advised thank you |
| The secondary objective—identifying factors associated with emergency contraceptive  use—is not clearly stated in the objectives. This should be made explicit in the  introduction and reflected throughout the manuscript. | Thank you very much for this observation The researchers aimed to ascertain whether knowledge and attitudes were associated with practices. To achieve this, both bivariate and multivariate analyses were conducted. However, the main objective of the study was to assess the knowledge, attitudes, and practices related to emergency contraceptive use. Putting it in the title and the main objective of the study is misleading we didn’t comprehensively collect data on other factors that would better achieve this objective |
| 2. Measurement and Cut-Off Clarity  o In line 167, the manuscript mentions that scores above 13 represent &quot;good knowledge.&quot; However, this is confusing, as the phrasing implies that a score of 13 is neither good nor poor. Please clarify the cutoff and specify the direction of the scale (i.e., does a higher score indicate better knowledge or attitude?). | This concern has been addressed by clarifying that a score of 13 and above was used as the cutoff for good knowledge. Since correct answers were coded with higher values, achieving a higher total score directly reflects a higher level of knowledge among respondents. This was done by similar studies |
| o Additionally, it is not clear whether this cutoff was validated or arbitrarily chosen by the authors. If it is author-defined, this should be clearly acknowledged with justification. | This has been addressed and justification for the cut off have been added |
| 3. Sample Size and Response Rate  o Lines 199–200 state that the calculated sample size was 600, but only 585 participants were recruited. However, this does not constitute a response rate. A response rate refers to the number of respondents relative to the number invited or contacted. If only 585 were contacted, the study did not reach its intended sample size, and this should be transparently described. | Thank you for this observation. It is true that the study did not reach the intended sample size. This has also been transparently described in the study. And therefore the response rate of the study was not 100% but rather 97.5% |
| 4. Results Structure and Flow  o The structure of the results section should better align with the stated objectives. First, describe the characteristics of the sample, followed by knowledge, attitudes, and emergency contraceptive use. Only then should results pertaining to associated factors (i.e., the secondary objective) be presented. | We appreciate the reviewer’s suggestion regarding restructuring the results section. However, in our study, practice was assessed using only one question about previous use of emergency contraceptive pills in the last 30 days. Given this, we felt it was more appropriate to report practice as a percentage in the text before the first table, rather than creating a separate section. |
| 5. Variables and Analyses  o In the &quot;Study Variables&quot; section (line 163), please explicitly list the variables considered for the multivariate analysis.  o It is unclear which covariates were included in the final model. Please specify these in the results section when presenting the multivariable analysis. | All the variables in the socio demographics and the overall knowledge and attitude were included in the multivariate analysis. This has instead been added to the analysis section of the study |
| 6. Terminology and Consistency  o Ensure consistent use of terms throughout the manuscript. For instance, the methods section uses “good knowledge vs poor knowledge,” but the results refer to “knowledgeable vs not knowledgeable.” Consistency is essential, particularly if the categories are author-defined.  o Spelling and typographical errors should be corrected, such as &quot;un married&quot; (line 241) which should be &quot;unmarried,&quot; and the double period on line 233. | Thank you for this observation. The manuscript has been revised to only include good knowledge and poor knowledge |
| 7. Table and Figure Presentation  o Table titles should appear directly above the corresponding tables. For example, Table 5  is mentioned in the text before it appears, creating confusion. | Thank you for this observation. it has been addressed as advised |
| o Table 4 is labeled as referring to knowledge, but the accompanying text addresses  attitudes. This inconsistency should be corrected. | This has been addressed thank you |
| 8. Interpretation of Attitudinal Data  o Line 230 refers to neutrality regarding the effectiveness of emergency contraceptives. It is unclear whether this is measuring belief in efficacy or safety. The phrasing should be revised to clarify the item participants responded to (e.g., “The majority of respondents were neutral in response to the statement: ‘Emergency contraceptives are safe and effective.’”). | Thank you for this observation the whole paragraph 230-238 has been rewritten as advised |
| 9. Discussion and Framing  o The discussion begins by emphasizing usage and associated factors, but the primary objective is to assess knowledge, attitudes, and use. Please realign the discussion to reflect the stated goals. | This has been addressed as advised and factors associated has been removed as the primary objective |
| o The phrase “individual factors” (line 254) is used in the plural, yet only one factor (marital status) appears significant. This should be corrected. | This has been removed from the paragraph |
| o Lines 271–286 repeat content from lines 251–270. Please revise to avoid redundancy. | Thank you so much for this observation. This was an error and has been rectified |
| 10. Conclusion and Bias Concerns  o The conclusion (line 297) suggests that non-use of emergency contraception is due to access limitations. This is an overgeneralization and could reflect bias. Alternative explanations—such as lack of perceived need, sexual inactivity, or personal preference—should be acknowledged.  o Similarly, line 352 suggests that married women use regular contraception due to planned sexual activity. Yet, if your inclusion criteria excluded those using regular contraceptives, this reasoning may not apply. | Thank you for this observation. it is very true and. the authors have revised the paragraph as advised |
| 11. Ethical Reporting and Safety Discussion  o The manuscript seems to advocate for proactive and regular use of emergency contraception. If this recommendation is made, information about the safety of frequent use must be included, ideally in the introduction and discussion sections. | This has been revised it has been made clear that “able to access emergency contraceptives whenever necessary to avoid unwanted or unplanned pregnancies” |
| 12. Formatting and Language  o The acknowledgements section (lines 364–365) is incomplete. o Careful proofreading is required to correct grammatical errors and awkward phrasing throughout. | This error has been rectified and the section has been completed |
