# Supplementary material for: Knowledge, attitudes and practices towards emergency contraceptive pill use among women of reproductive age in Lira District, Northern Uganda: A cross-sectional study
Source: PLoS One. 2026 Mar 26;21(3):e0346151. doi: 10.1371/journal.pone.0346151 (PMC13020816; doi:10.1371/journal.pone.0346151)
Supplement: Attachment — Submitted filename: Comments6 .docx [file pone.0346151.s005.docx]

We sincerely thank the Editor and Reviewer 1 for their careful review of our manuscript and for the constructive comments provided. We greatly appreciate the time and effort invested in evaluating our work. The feedback has been invaluable in improving the clarity, scientific rigor, and overall quality of the manuscript.

In response to the comments, we have thoroughly revised the manuscript. Particular attention was given to improving the quality of written English, strengthening the discussion section, eliminating repetition, clarifying methodological details, and refining the presentation of results. We have also carefully addressed issues related to statistical interpretation, formatting consistency, and terminology throughout the manuscript.

All comments have been addressed point-by-point below, and corresponding revisions have been incorporated into the revised manuscript. We hope that the changes made have satisfactorily addressed the concerns raised and improved the manuscript to meet the journal’s standards.

| **Comments** | **Response** |
| --- | --- |
| **Editor** |  |
| 1. concerns regarding the quality of written English and the manuscript will benefit from further editing. For instance, line 15 - spelling of "College" written as "Collage" | This has been addressed as reviewers have raised the same concerns, we have done our best to proof read the manuscript and ensure that that the English is improved |
| Rewrite the discussion, in particular the first paragraph. Summarize the key findings of your study as it concerns the objectives, and interpret it. | The discussion section has been rewritten to address all the comments raised |
| Avoid repeating the result as earlier presented unless necessary. | All the repeated results have been removed unless necessary |
| Highlight implication of your findings to policy makers and healthcare professionals | We have directed our implications to the policy makers and health care professionals especially the factors associated |
| Under the limitation, address recall bias. | The limitations paragraph has been rewritten and all the limitations of the study have been elaborated. |
| **Reviewer 1** |  |
| Line 39: The full stop after the SPSS version should be removed | This has been addressed as advised Thank You |
| Line 43 and 44: the figures in there should be in parenthesis eg (65.98%) | Thank you very much for this comment it has been addressed |
| Line 60: Remove the space between year and the full stop and bring the space rather after the full stop | Thank you for this observation. This has been addressed |
| Line 61: unprotected is one word. Kindly correct this and similar ones in the entire manuscript. | This has been addressed thank you |
| Line 90: remove the space before the full stop. Kindly correct this and similar ones in the entire manuscript. | Thank you for this observation. The comment is addressed |
| Line 124: Kindly display the formula before assigning values to the variables in the formula. | The formula has been added to the manuscript |
| Line 126: the sentence beginning after the full stop should start with a capital letter (The resulting …..). Kindly correct such and similar ones in the entire manuscript. Line 259 also refers | Thank you for this observation this has been corrected |
| Line 126: the sentence beginning after the full stop should start with a capital letter (The resulting …..). Kindly correct such and similar ones in the entire manuscript. | Thank you for this observation this correction has been addressed |
| Line 259 also refers | This has also been addressed as advised thank you |
| Line 154: unintended is one word. Kindly correct such and similar words in the entire manuscript. | Thank you for this observation. This has been corrected |
| Line 171: if the variables in the Likert scale started with capital letters, then for consistency’s sake, strongly disagree should also follow the same pattern. Please correct all such in the entire manuscript. Refer line 206 as well for Residence, table 3 for ‘some emergency…..’ | This has been addressed as advised thank you |
| Line 214/215: are the authors suggesting that high doses of POP and COC cannot be used for emergency contraception? Authors should refer to the YUZPE regimen for EC. Kindly address the intent of that statement and let it be clearer. | This not correctly written and has been revised to ensure that it is correct and the Yuze regiment has been noted |
| Table 5: Un married to be unmarried. | Thank you for this observation. This has been addressed as advised |
| Line 259: address the full stop and continue with small letter. Ie ‘Kenya. their’ kindly correct. | Thank you for this comment this error has been corrected |
| Line 326: un intended should be one word | Thank you for this comment the error has been corrected |
| Line 328: change Negative to negative. improve the sentence. The figure cannot be at the end of the sentence in this current construct. | This has been addressed as advised thank you |
| Line 334: should be resource constrained area not areas | This has been rewritten |
| Line 296- 306 content has been repeated in Line 307 -318. This was earlier out to authors in Review 1 but the Authors are yet to address it. | Thank you very much for understanding and reemphasising this it has been corrected and the repetition removed |
| Line 323 -325: sentence should be improved | Thank you, the whole paragraph has been improved, |
| Line 330: change the ‘if’ to ‘of’ this study | This had been corrected as advised |
| I suggest the authors thoroughly proofread the document and improve on the grammar and concordances of the sentences. | We have done our best to proof read the manuscript Thank you for the advise and the review |
