# Supplementary material for: Knowledge, attitudes and practices towards emergency contraceptive pill use among women of reproductive age in Lira District, Northern Uganda: A cross-sectional study
Source: PLoS One. 2026 Mar 26;21(3):e0346151. doi: 10.1371/journal.pone.0346151 (PMC13020816; doi:10.1371/journal.pone.0346151)
Supplement: Attachment — Submitted filename: Comments 7.docx [file pone.0346151.s006.docx]

Dear Editor and Reviewers,

We sincerely thank you for your careful review of our manuscript entitled “Knowledge, Attitudes, and Practices Towards Emergency Contraceptive Pill Use Among Women of Reproductive Age in Lira District, Northern Uganda.” We greatly appreciate your constructive comments and valuable suggestions, which have helped us improve the clarity and quality of the manuscript. We have carefully revised the manuscript and addressed all the comments raised. A detailed point-by-point response is provided below, and the corresponding revisions have been made in the manuscript.

We thank you again for your helpful feedback.

| **Comments** | **Response** |
| --- | --- |
| **1. under the abstract, rewrite lines 31-32 or delete some redundant words** | Thank you for this comment. We revised the relevant sentences in the abstract to remove redundant wording and improve clarity and conciseness. |
| **2. Line 38; rewrite this or delete quantitative methods in the sentence** | Thank you for this comment. The sentence was revised and the phrase “quantitative methods” was removed for clarity. |
| **3. Line 45; state the mean age with the SD** | Thank you for this observation. We revised the results section of the abstract to report the mean age together with the standard deviation as follows: *“The mean age of participants was 28.01 ± 7.6 years.”* |
| **4. Line 54-55; doesn't make sense. Rewrite or paraphrase it** | Thank you for this comment. We rephrased the conclusion in the abstract for clarity and readability. |
| **5. Under background, line 76. Delete "relatively" from the statement** | Thank you. The word “relatively” was deleted as suggested. |
| **6. Line 84/85, reword the statement and reference low awareness** | Thank you for this important observation. We revised the statement to emphasize the low awareness of emergency contraception among women in Uganda and supported it with the appropriate reference. |
| **7. line 90; rewrite this statement either address contraceptive prevalence rates or non use rate to avoid confusion** | Thank you. We revised the statement to consistently refer to the contraceptive non-use rate in order to avoid confusion. |
| **8. Line 95; "the findings will..." should read "the findings may.."** | Thank you. The statement was revised to read “the findings may...” to reflect appropriate caution in interpretation. |
| **9. Line 101; delete "quantitative methods" from the statement** | Thank you. The phrase “quantitative methods” was deleted and the sentence was revised accordingly. |
| **10. Reconcile line 90 & line 108. Which one is correct?** | Thank you for this observation. We reconciled the two statements and used the contraceptive non-use rate consistently throughout the manuscript to avoid contradiction. |
| **11. Under the setting, line 100, provide information concerning occupation and religion of the residents or inhabitants as they influence utilization of EC** | Thank you. We added information on the main occupations and religious composition of residents in Lira District, and briefly linked these contextual factors to possible influence on the utilization of emergency contraception. |
| **12. line 139, the multistage sampling table presented should reflect the estimated sample size of 600 not 585** | Thank you for this comment. An additional column was included to show the planned number of respondents to be selected from each village based on the estimated sample size of 600, alongside the final achieved sample of 585. |
| **13. Line 141- Data collection: who were the research assistants that administered the questionnaire? were they trained? By whom? For how long? what's their gender?** | Thank you for this important comment. We revised the data collection section to clarify that data were collected by five female research assistants who were university graduates. We also added that they received a two-day training conducted by the research team together with a member of the Research Ethics Committee. |
| **14. Rewrite line 145** | Thank you. The sentence was rewritten to improve clarity and flow, and now better explains the pretesting of the questionnaire in a comparable setting. |
| **15. Line 194, how was privacy ensured during administration of the questionnaire?** | Thank you for this comment. We added a statement in the ethical considerations section explaining that interviews were conducted in a private setting within or near the participant’s home, out of hearing range of other household members, and only the participant and research assistant were present. |
| **16. Under results, line 205. If 585 respondents were approached and they all participated, the response rate is 100 %. Review this.** | Thank you for this observation. We reviewed this section and clarified the wording. The response rate remains 97.5% because the estimated sample size was 600, but 585 respondents were ultimately enrolled and participated. |
| **17. line 162 - study variables. State clearly who gets 1,2, and 3 points. If the correct answer attracts 3 points, then some respondents may get above 13 without adequate knowledge using your criterion. This calls for concern, and may send a wrong signal.** | Thank you for this valuable comment. We revised the study variables section to clearly state that correct responses were assigned 3 points, partially correct responses 2 points, and incorrect or “don’t know” responses 1 point. We also clarified the score range and explained the rationale for the cutoff used to categorize knowledge levels. |
| **18. Under discussion, line 268. Rewrite the statement "the result of our study".....** | Thank you. The wording was revised for better academic tone and clarity. |
| **19. Group the utilization of EC in this study and compare with similar studies in the same paragraph. Same applies to knowledge.** | Thank you for this suggestion. We reorganized the discussion so that findings on emergency contraceptive utilization are discussed together and compared with similar studies in one paragraph. We did the same for knowledge findings in a separate paragraph. |
| **20. The lines 293 - 300 should come much earlier in the discussion.** | Thank you. We moved this section earlier in the discussion so that the explanation regarding recent use versus lifetime use appears immediately after the comparison of utilization findings. |
| **21. No conclusion or recommendation based on the findings. It is important to distinguish between utilization of EC and access to it, especially in making recommendations.** | Thank you for this important observation. We revised the discussion to clearly distinguish between utilization and access to emergency contraception. We also strengthened the concluding part of the discussion by adding clear implications and recommendations based on the findings, with careful wording to avoid conflating use with access. |
